# Socioemotional Engagement with Grandchildren Is Associated With Benefits for Grandparents

**DOI:** 10.1093/geroni/igaf122.1592

**Published:** 2025-12-31

**Authors:** Claire Growney, Thomas Oltmanns, Patrick Hill, Ryan Bogdan, Laura Carstensen

**Affiliations:** Stanford University, Stanford, California, United States; Washington University in St. Louis, St. Louis, Missouri, United States; Washington University in St. Louis, Saint Louis, Missouri, United States; Washington University in St. Louis, St. Louis, Missouri, United States; Stanford University, Stanford, California, United States

## Abstract

As life expectancy has increased, grandparenting has become a normative social role, yet its implications for older adults’ well-being remain understudied. The present study examined well-being as a function of the amount and type of engagement grandparents have with their grandchildren. Participants (N = 1,002) aged 66–79 in the St. Louis Personality and Intergenerational Network (SPIN) study completed questionnaires about physical health, health practices, loneliness, and subjective memory and indicated whether they were grandparents. The 533 who identified as grandparents also answered questions about their involvement with grandchildren and related emotional experiences. There were minimal differences between grandparents and non-grandparents. However, among grandparents, those who were relatively more engaged socially and emotionally with their grandchildren reported less loneliness, more engagement in healthful behaviors, and better subjective memory. Socioemotional engagement with grandchildren was also associated with more positive and less negative emotional experience. Grandparents who reported providing relatively high levels of both instrumental support and socioemotional engagement reported better overall physical health-related quality of life. In contrast, providing relatively more instrumental support for grandchildren was associated with higher levels of loneliness and unrelated to healthful behavior engagement, subjective memory, or emotional experiences. Social and emotional ties to grandchildren are associated with cognitive, physical, social functioning among older adults, highlighting the importance of considering potential benefits and costs of grandparental involvement. We suggest that socioemotional engagement with grandchildren represents a particularly meaningful social connection, fostering a sense of purpose.

